# miR-129-5p Regulates the Immunomodulatory Functions of Adipose-Derived Stem Cells via Targeting Stat1 Signaling

**DOI:** 10.1155/2019/2631024

**Published:** 2019-10-21

**Authors:** He-Yang Zhang, Yu-Han Wang, Yan Wang, Yan-Nv Qu, Xiao-Hui Huang, Hui-Xin Yang, Chang-Ming Zhao, Youdi He, Si-Wei Li, Jin Zhou, Changyong Wang, Xiao-Xia Jiang

**Affiliations:** ^1^Department of Neural Engineering and Biological Interdisciplinary Studies, Institute of Military Cognition and Brain Sciences, Academy of Military Medical Sciences, 27 Taiping Road, Haidian District, Beijing 100850, China; ^2^Department of Animal Science and Biotechnology, College of Agriculture and Life Science, Chungnam National University, 220 Gung-dong, Yuseong-gu, Daejeon 305-764, Republic of Korea; ^3^Department of Neurology, Beijing Chaoyang Hospital, Capital Medical University, Beijing 100020, China; ^4^Anhui Medical University, Hefei, 230032 Anhui, China

## Abstract

Adipose-derived stem cells (ASCs) have become one of the most promising stem cell populations for cell-based therapies in regenerative medicine and for autoimmune disorders owing to their multilineage differentiation and immunomodulatory capacities, respectively. One advantage of ASC-based therapy lies in their immunosuppressive potential. However, how to get ASCs to provide consistent immunosuppression remains unclear. In the current study, we found that miR-129-5p was induced in ASCs treated with inflammatory factors. ASCs with miR-129-5p knockdown exhibited enhanced immunosuppressive capacity, as evidenced by reduced expression of proinflammatory factors, with concurrent increased expression of inducible nitric oxide synthases (iNOS) and nitric oxide (NO) production. These cells also had an increased capacity to inhibit T cell proliferation *in vitro*. ASCs with miR-129-5p knockdown alleviated inflammatory bowel diseases and promoted tumor growth *in vivo*. Consistently, ASCs that overexpressed miR-129-5p exhibited reduced iNOS expression. Furthermore, we show that miR-129-5p knockdown in ASCs results in hyperphosphorylation of signal transducer and activator of transcription 1 (Stat1). When fludarabine, an inhibitor of Stat1 activation, was added to ASCs with miR-129-5p knockdown, iNOS mRNA and protein levels were significantly reduced. Collectively, these results reveal a new role for miR-129-5p in regulating the immunomodulatory activities of ASCs by targeting Stat1 activation. These novel insights into the mechanisms of ASC immunoregulation may lead to the consistent production of ASCs with strong immunosuppressive functions and thus better clinical utility of these cells.

## 1. Introduction

Adipose-derived stem cells (ASCs) have proven to be one of the most promising stem cell populations to date [1, 2]. ASCs can easily be obtained from adipose tissue through minimally invasive procedures such as liposuction [3]. In addition to their pluripotent differentiation potential, which contributes to high expectations for their use in regenerative medicine, the discovery of ASC immunomodulatory properties allows ASC-based therapies to cross the bridge from the field of regenerative medicine to autoimmunity [4, 5].

Similar to bone marrow-derived mesenchymal stem cells (MSCs), allogeneic ASCs are immune privileged and have immunomodulatory properties [4]. ASCs can reduce the proliferation and regulate the function of activated immune cells *in vitro* through cell-cell interaction and paracrine signaling, affecting both innate and adaptive immunity [6]. ASCs have also demonstrated therapeutic potential in numerous immune-mediated disorders *in vivo* in both animal models and clinical studies, including systemic sclerosis, graft-versus-host disease (GvHD), and chronic inflammatory autoimmune diseases [7–10]. A series of factors and molecules produced by ASCs have been shown to be critical for their immunoregulatory functions, such as nitric oxide (NO) [11], indoleamine 2,3-dioxygenase (IDO) [12], prostaglandin (PG) E2 [12, 13], transforming growth factor *β* (TGF-*β*) [14], and interleukin- (IL-) 10 [14, 15].

The immunomodulatory effects of ASCs are not always immunosuppressive. On parallel with ASCs, several studies have shown that MSCs can become highly immunosuppressive with an MSC2 phenotype but can enhance immune responses when endowed with an MSC1 phenotype [16]. Transcription factors such as suppressor of cytokine signaling 1 (SOCS1) [17] and the ubiquitin-modifying enzyme A20 [18], as well as epigenetic factors such as Mysm1 [19], miR-155 [20], and miR-150 [19], have been reported to play important roles in the immunomodulatory capacity of MSCs. An advantage of MSC-based therapy lies in their immune plasticity. However, the proximity and short time frame of their immunosuppressive effects limit their applicability in clinical trials [21]. Therefore, to understand how to impose consistent immunosuppression, for example, in the case of autoimmune disorders, requires further mechanistic studies.

miRNAs play a critical role in the regulation of immune responses. miR-129-5p has shown great promise as a potential therapeutic agent because of its targeting of important genes associated with apoptosis, cell cycle, and chemoresistance [22–24]. The functional significance of miR-129-5p in cancer has been investigated in detail in recent years [25–27]. However, its role in ASC immunoregulatory function has yet to be investigated.

In the current study, we showed that ASCs with a miR-129-5p knockdown are highly immunosuppressive, as exhibited by a decrease in the expression of tumor necrosis factor-*α* (TNF*α*), interferon gamma (IFN*γ*), and IL-1*β* with accompanying increased expression of inducible nitric oxide synthase (iNOS). Moreover, knockdown of miR-129-5p in ASCs enhanced their ability to inhibit T cell proliferation *in vitro* and alleviated inflammatory bowel disease while promoting tumor growth *in vivo*. Further mechanistic studies revealed that miR-129-5p regulates the immunomodulatory capacity of ASCs partly via enhancing Stat1 phosphorylation. Taken together, these results reveal a novel role of miR-129-5p in regulating the immunomodulatory properties of ASCs.

## 2. Materials and Methods

### 2.1. Animals

Groups of 3-4-week-old and 8-12-week-old C57BL/6 mice were obtained from the Laboratory Animal Center of the Academy of Military Medical Sciences of China (Beijing). In all experiments, age- and sex-matched wild-type (WT) littermates were used for controls. Mice were maintained in a pathogen-free barrier facility. All animal experiments were performed according to the Guide for the Care and Use of Laboratory Animals and were approved by the Institute of Military Cognition and Brain Sciences. The institutional Ethics Review Committee for Animal Experimentation approved all experimental protocols.

### 2.2. ASC Isolation and Culture

Three-to-four-week-old C57BL/6 mice were euthanized by cervical dislocation and sterilized in 75% ethanol for 5 min. ASCs were obtained from inguinal subcutaneous adipose tissue with the abdomens facing up. The collected tissue was rinsed with phosphate buffered saline (PBS) and minced, followed by digestion with 1 mg/mL collagenase type IV (Sigma-Aldrich, St. Louis, MO, USA) and 1 mg/mL dispase (Sigma-Aldrich) for 35 min at 37°C with agitation. The cells were then added to an *α*-minimum essential medium (*α*-MEM) (Gibco, Carlsbad, CA, USA) containing 10% fetal bovine serum (FBS; Gibco) to stop digestion and filtered through a 40 *μ*m cell strainer (Biologix, Lenexa, KS, USA) to generate single-cell suspensions. After centrifugation at 400 g for 5 min, cells were resuspended in an *α*-MEM containing 10% FBS and cultured at 37°C with 5% CO_2_. Upon 80-90% confluence, cells were subcultured at a ratio of 1 : 3.

### 2.3. Carboxyfluorescein Diacetate Succinimidyl Ester Labelling

Splenocytes were isolated from C57BL/6 mice (8-12 weeks old). CD3^+^ T cells were selected using a CD3*ε* MicroBead Kit (Miltenyi Biotec, Bergisch Gladbach, Germany) and then labeled with 5 *μ*M carboxyfluorescein diacetate succinimidyl ester (CFSE, Invitrogen, Carlsbad, CA, USA) for 7 min at 4°C. Labeling was terminated according to the manufacturer's protocol. After washing, cells were activated with 50 ng/mL phorbol myristate acetate (PMA) and 1 *μ*g/mL ionomycin (Sigma-Aldrich) for 16 h and then cocultured with or without ASCs for 48 h. Cell division, as indicated by reduction of fluorescence intensity, was analyzed by flow cytometry.

### 2.4. Quantitative RT-PCR

Total RNA was extracted with TRIzol (Sigma-Aldrich) and 1 *μ*g of RNA reverse transcribed into cDNA with a reverse transcriptase kit (Toyobo, Osaka, Japan). cDNA was then used as a template in quantitative PCR with SYBR Green (Toyobo) to determine specific gene expression. Total mRNA was normalized to endogenous GAPDH mRNA. Primer pairs were as follows: iNOS (ID: NM_010927.4) CAGCTGGGCTGTACAAACCTT (forward) and CATTGGAAGTGAAGCGTTTCG (reverse) (product length: 95); TNF*α* (ID: NM_013693.3) GATGGGTTGTACCTTGTCTACT (forward) and CTTTCTCCTGGTATGAGATAGC (reverse) (product length: 112); IFN*γ* (ID: NM_008337.4) GGTCAACAACCCACAGGTC (forward) and GACTCCTTTTCCGCTTCCT (reverse) (product length: 101); IL-1*β* (ID: NM_008361.4) CATTAGACAACTGCACTACAGG (forward) and GTTCTCCTTGTACAAAGCTCAT (reverse) (product length: 146); and GAPDH (ID: NM_008084.3) ACAATGAATACGGCTACAG (forward) and GTCCAGGGTTTCTTACTC (reverse) (product length: 77). miR-129-5p determination was followed by the instruction of products (B532451 for cDNA synthesis, B532461 for PCR) from Sangon Biotech (Shanghai, China). The levels of gene expression were calculated by relative quantification using GAPDH or snU6 as the endogenous reference genes. The *Δ*Ct value was calculated by subtracting the Ct value for GAPDH or for snU6 from the Ct value for the gene of interest.

### 2.5. Lentivirus Production and Transduction

Recombinant lentiviral vectors encoding for an inhibitor of miR-129-5p or a mimic and control lentiviral vectors were purchased from GeneChem (Shanghai, China). ASCs were transduced as we have previously described [19].

### 2.6. Induction of Acute Colitis

Acute colitis was induced in C57BL/6 mice by administering 3% dextran sodium sulfate (DSS; molecular weight 40,000 Da; Sigma-Aldrich) from day 0 to day 7 in drinking water. On day 1, ASCs transduced with lentivirus encoding for an inhibitor of miR-129-5p (named miR-129-5p inhibitor) or control (named NC) lentivirus were injected intraperitoneally into DSS-treated animals. Colitis severity was assessed daily by scoring (0-4) of the clinical disease activity through evaluation of stool consistency, presence of fecal blood, and weight loss. Mice with acute colitis were euthanized on day 8. The entire colon was removed from the caecum to the anus, and colon length and weight were measured as indirect markers of inflammation. The macroscopic colonic damage score was assessed based on the grade of tissue adhesion, presence of ulceration, and wall thickness.

### 2.7. Mouse Melanoma Model

B16-F0 murine melanoma cells (CRL-6322, ATCC, Manassas, VA, USA) were expanded in complete Dulbecco's MEM (DMEM) *in vitro*. Each mouse was injected with 5 × 10^5^ B16-F0 cells in 100 *μ*L PBS intramuscularly on the left thigh, with or without coinjection of miR-129-5p inhibitor- or NC-transduced ASCs (1 × 10^6^ per mouse). Mice were observed daily and euthanized when tumors began to significantly affect mobility. Melanoma tumors were then excised and weighed. Each experimental group included at least five mice.

### 2.8. Immunofluorescence Staining

For immunofluorescence, cells were fixed in 4% formaldehyde for 20 min at room temperature, permeabilized with 0.3% Triton X-100 in PBS for 10 min, and then incubated with 2% BSA and 0.05% sodium azide in PBS for 1 h at room temperature to block nonspecific antibody binding. Subsequently, cells were incubated with primary antibodies against the cell markers of interest overnight at 4°C. Cells were then washed three times for 10 min with PBS and incubated for 1 h at room temperature with an Alexa Fluor 594-conjugated secondary antibody (Invitrogen). Then, the cells were counterstained with 4′,6-diamidino-2-phenylindole (DAPI) in Dulbecco's PBS (DPBS; Sigma-Aldrich) and analyzed under a fluorescence microscope (Nikon AZ-100 multipurpose microscope).

### 2.9. Western Blot

Cells were lysed with lysis buffer to isolate proteins, and then the same amount of protein samples, 20 *μ*g for each well, was subsequently separated via 12% sodium dodecyl sulfate-polyacrylamide gel electrophoresis (SDS-PAGE), then transferred to 0.45 *μ*m polyvinylidene fluoride (PVDF) blotting membranes. The membranes were blocked with 5% nonfat dry milk in PBS (blocking solution) for 1 h, probed with primary antibodies against the proteins of interest in blocking solution overnight at 4°C, washed, and then incubated with HRP-conjugated secondary antibodies in blocking solution for 1 h at room temperature. Finally, enhanced chemiluminescence substrate (Thermo Fisher, Waltham, MA, USA) was added to the membranes and the proteins were assayed according to the manufacturer's instructions. All antibodies were purchased from Cell Signaling Technology (Beverly, MA, USA).

### 2.10. Detection of NO

ASCs were stimulated with TNF*α* plus IFN*γ* for 24 h. NO was detected with a modified Griess reagent (Sigma-Aldrich) and assayed according to the manufacturer's instructions.

### 2.11. Statistical Analysis

All data were analyzed with Prism 5.0 software (GraphPad Software, San Diego, CA, USA) and are presented as the means ± standard deviations (SDs). Statistical significance was assessed by unpaired two-tailed Student's *t*-tests (^∗^*p* < 0.05; ^∗∗^*p* < 0.01).

## 3. Results

### 3.1. miR-129-5p Was Induced in ASCs

**Figure 1:**
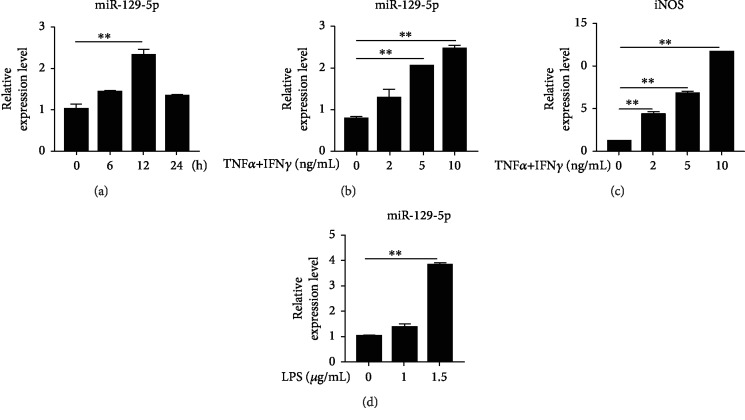
Inflammatory factors induce miR-129-5p expression in ASCs. ASCs were treated with 10 ng/mL TNF*α* plus 10 ng/mL IFN*γ* for the indicated amount of time (a) or treated with TNF*α* plus IFN*γ* for 12 h at increasing concentrations (b, c) and then collected in TRIzol. miR-129-5p and iNOS mRNA levels were determined by quantitative RT-PCR. (d) ASCs were treated with LPS for 12 h at increasing concentrations, and miR-129-5p levels were examined by quantitative RT-PCR. ^∗∗^*p* < 0.01.

miRNAs are critical in the regulation of immune responses. As immunoregulatory cells, ASCs have gained great interest in the fields of regenerative medicine and autoimmunity. We previously found that the expression of miR-129-5p was among the miRNAs most significantly increased in MSCs treated with TNF*α* and IFN*γ* as assayed by miRNA microarrays (data not shown). To further examine the effect of miR-129-5p on the immunomodulatory function of ASCs, the expression of miR-129-5p was analyzed in ASCs treated with inflammatory cytokines. Upon TNF*α* and IFN*γ* stimulation, miR-129-5p expression exhibited the most significant increase at 12 h (Figure 1(a)), was found to be dose-dependent (Figure 1(b)), and was accompanied by increased expression of iNOS (Figure 1(c)) and inflammatory cytokines (data not shown). Additionally, when ASCs were treated with 1.5 *μ*g/mL LPS, miR-129-5p expression was significantly increased (Figure 1(d)). These data indicate that miR-129-5p may be involved in the immunomodulatory activity of ASCs.

### 3.2. ASCs with miR-129-5p Knockdown Exhibit Enhanced Immunosuppressive Capacity *In Vitro*

**Figure 2:**
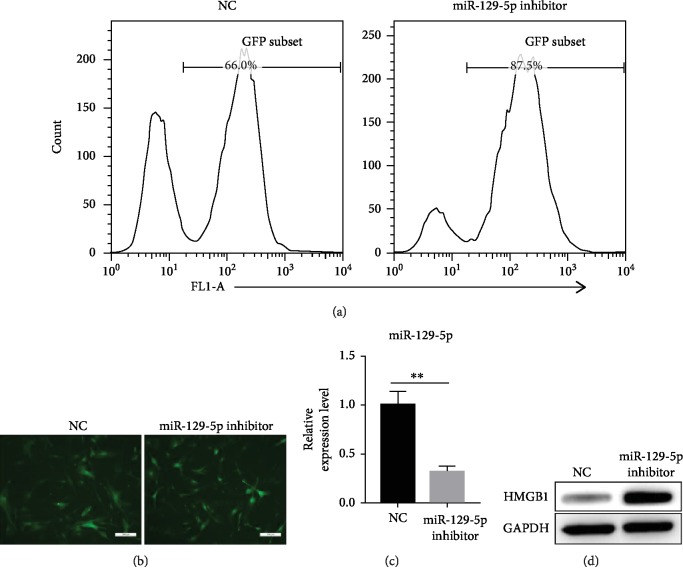
miR-129-5p knockdown in ASCs by lentiviral transduction. ASCs were isolated from C57BL/6 mice and were transduced with control lentivirus containing GFP (NC) or lentivirus containing miR-129-5p inhibitor and GFP. GFP expression was examined by flow cytometry (a) and fluorescence microscopy (b). (c) miR-129-5p levels in NC- and miR-129-5p inhibitor-transduced ASCs were determined by quantitative RT-PCR. (d) HMGB1 expression was examined by western blot. Scale bar: 200 *μ*m. ^∗∗^*p* < 0.01.

**Figure 3:**
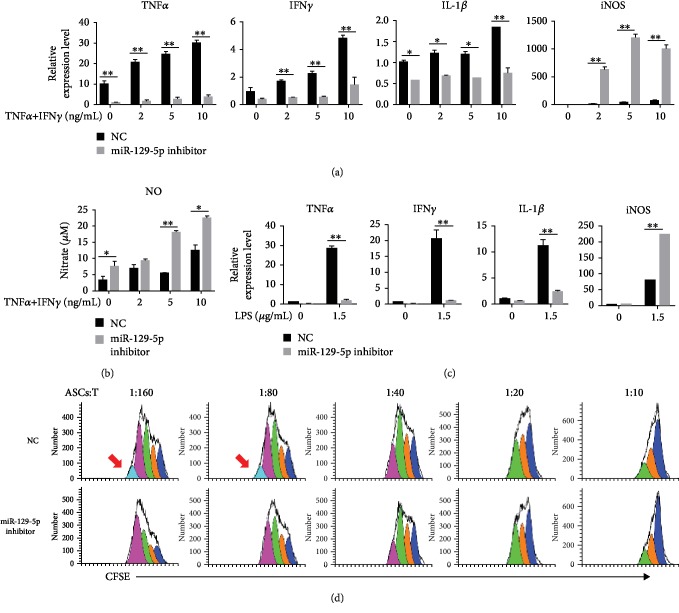
iNOS expression in and inhibition of T cell proliferation by ASCs with miR-129-5p knockdown. Both NC- and miR-129-5p inhibitor-transduced ASCs were treated with TNF*α* plus IFN*γ* for 12 h at increasing concentrations. (a) mRNA levels of inflammatory cytokines in NC- and miR-129-5p inhibitor-transduced ASCs were determined by quantitative RT-PCR. (b) After treatment with TNF*α* plus IFN*γ* for 24 h, the amount of nitric oxide in the supernatant of NC- and miR-129-5p inhibitor-transduced ASCs was examined by a Griess test. (c) Both NC- and miR-129-5p inhibitor-transduced ASCs were treated with LPS (1.5 *μ*g/mL) for 12 h. mRNA levels of inflammatory cytokines were then determined by quantitative RT-PCR. (d) CD3^+^ T cells isolated from murine spleens were labeled with CFSE and stimulated with PMA (50 ng/mL) plus ionomycin (1 *μ*g/mL) for 24 h and then cocultured with NC- or miR-129-5p inhibitor-transduced ASCs at different ratios (ASC : T cell). After 48 h, cells were analyzed by flow cytometry for T cell proliferation as indicated by reduced CFSE fluorescence intensity. ^∗^*p* < 0.05 and ^∗∗^*p* < 0.01.

To investigate the involvement of miR-129-5p in ASC immunomodulatory activity, ASCs were isolated and transduced with a lentiviral vector containing miR-129-5p inhibitor or control (NC) lentivirus. Transduction efficiency was examined by GFP expression via flow cytometry and fluorescence microscopy analyses (Figures 2(a) and 2(b)). Reductions in miR-129-5p expression in miR-129-5p inhibitor-transduced ASCs were determined by quantitative RT-PCR (Figure 2(c)). High mobility group protein 1 (HMGB1) is a direct target of miR-129-5p. High protein levels of HMGB1 in the miR-129-5p inhibitor-transduced group confirmed the knockdown of miR-129-5p (Figure 2(d)). To better characterize the function of miR-129-5p, inflammatory cytokine expression in ASCs transduced with miR-129-5p inhibitor or NC was analyzed. ASCs transduced with miR-129-5p inhibitor demonstrated lower expression of the inflammatory genes TNF*α*, IFN*γ*, and IL-1*β* and significantly higher expression of iNOS compared to controls upon stimulation with increasing amounts of TNF*α* plus IFN*γ* (Figure 3(a)). Correspondingly, ASCs transduced with miR-129-5p inhibitor exhibited significantly higher NO production compared to controls (Figure 3(b)). When treated with LPS, ASCs transduced with miR-129-5p inhibitor also showed lower expression of the inflammatory genes TNF*α*, IFN*γ*, and IL-1*β* and higher expression of iNOS compared to NC-transduced ASCs (Figure 3(c)). T cell proliferation is commonly used as an immune response model, in which reductions of CFSE fluorescence intensity are measured to determine the degree of cell division. As shown in Figure 3(d), ASCs transduced with miR-129-5p inhibitor or NC both inhibited T cell proliferation at different ASC : T cell ratios, though ASCs transduced with miR-129-5p inhibitor did so to a greater extent as evidenced by less reduction in CFSE fluorescence intensity at lower ASC : T cell ratios.

### 3.3. miR-129-5p Knockdown in ASCs Alleviated Dextran Sulfate Sodium-Induced Colitis but Promoted Tumor Growth *In Vivo*

**Figure 4:**
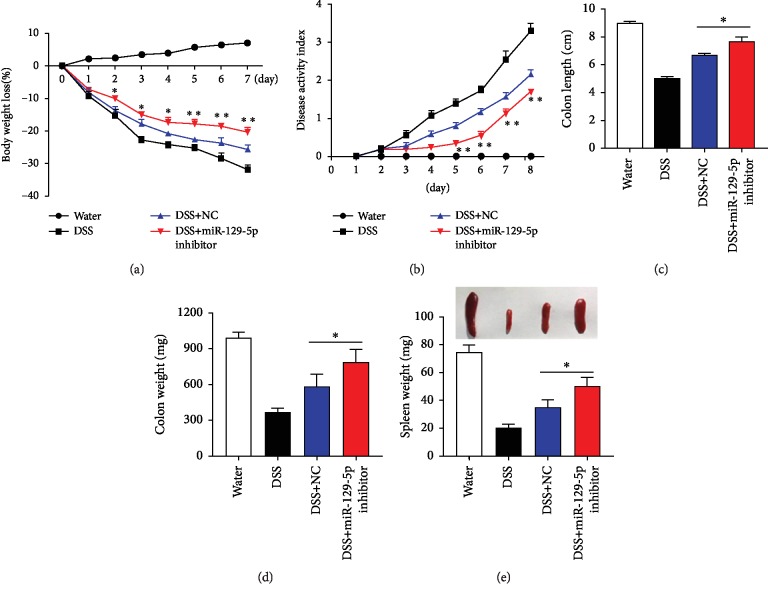
ASCs with miR-129-5p knockdown alleviate DSS-induced colitis *in vivo*. (a) C57BL/6 mice received 3% DSS in drinking water from day 0 to day 8. ASCs (1 × 10^6^/mouse) were infused intraperitoneally on day 1. Body weight loss (a) and disease activity scores (b) were evaluated daily. Colon length (c), colon weight (d), and spleen weight ((e), bottom) were measured on day 8. Representative spleen pictures were taken on day 8 ((e), top). Control mice received no DSS in drinking water. *n* = 5 mice/group. Statistical comparisons were made between the DSS+NC and DSS+miR-129-5p inhibitor groups. ^∗^*p* < 0.05 and ^∗∗^*p* < 0.01.

Next, the physiological function of ASCs with miR-129-5p knockdown was investigated in an experimental murine model of acute colitis induced by oral DSS administration. After 8 days, mice receiving oral administration of 3% DSS exhibited a significant increase in the disease activity index, characterized by acute colitis, bloody diarrhea, and sustained weight loss (Figures 4(a)–4(e)). Subsequent treatment with ASCs transduced with miR-129-5p inhibitor or control (NC) increased survival rate, ameliorated total weight loss, reduced spleen weight loss, and improved disease severity. Consistent with the enhanced immunosuppressive capacity of ASCs observed in the *in vitro* T cell proliferation assay, ASCs with miR-129-5p knockdown alleviated DSS-induced colitis to a significantly greater extent than control (NC) ASCs (Figures 4(a)–4(e)).

**Figure 5:**
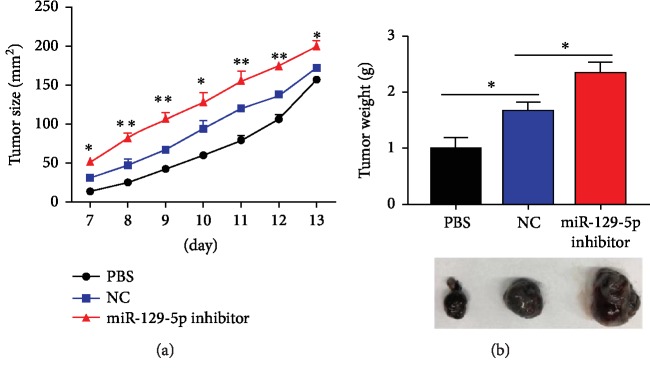
ASCs with miR-129-5p knockdown promote tumor growth *in vivo*. On day 1, 5 × 10^5^ B16-F0 cells were injected subcutaneously into the back of C57BL/6 mice, with or without coinjection of NC- or miR-129-5p inhibitor-transduced ASCs (1 × 10^6^ cells per mouse). After 7 days, tumor size was measured daily (a). On day 13, mice were euthanized and the tumors were weighed ((b), top), and a representative tumor from each group is shown ((b), bottom). Statistical comparisons were made between the NC and miR-129-5p inhibitor groups. ^∗^*p* < 0.05 and ^∗∗^*p* < 0.01.

Previous studies have shown that MSCs with enhanced immunosuppressive capacity promote tumor growth [16, 17]. In the current study, ASCs with miR-129-5p knockdown were found to be more immunosuppressive both *in vitro* and *in vivo* compared to control ASCs. Thus, a murine melanoma model was used to determine the effect of miR-129-5p knockdown in ASCs on tumor growth *in vivo*. B16-F0 melanoma cells were coadministered with ASCs transduced with miR-129-5p inhibitor or control (NC), and the resultant tumors were weighed after 13 days. Though control ASCs promoted tumor growth, ASCs with miR-129-5p knockdown were found to promote tumor growth much more significantly (Figures 5(a) and 5(b)).

### 3.4. Hyperphosphorylation of Stat1 in ASCs with miR-129-5p Knockdown

**Figure 6:**
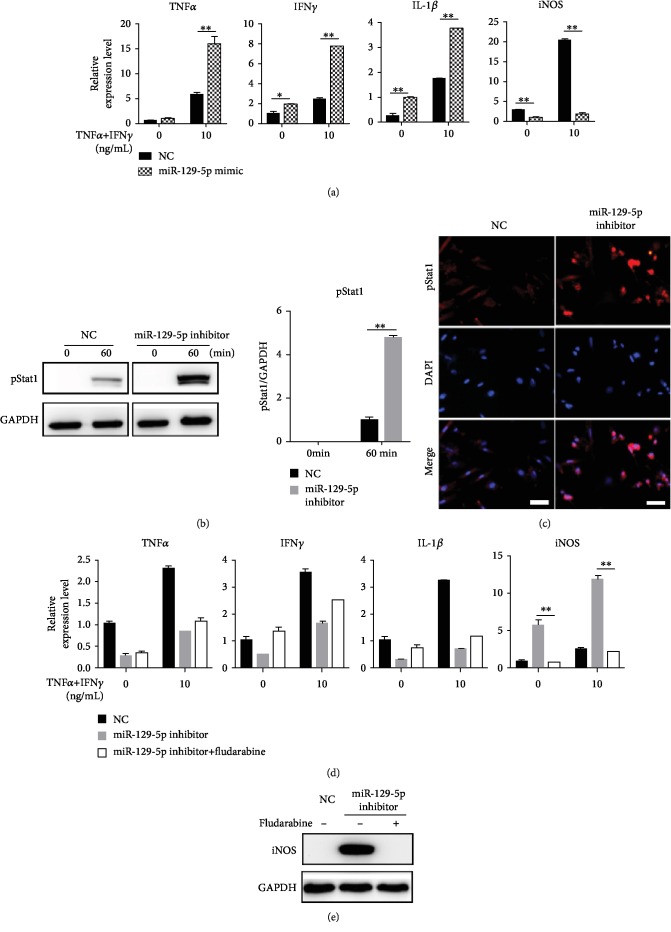
Enhanced expression of pStat1 in ASCs with miR-129-5p knockdown. (a) ASCs were isolated from C57BL/6 mice and were transduced with lentivirus containing GFP (NC) or miR-129-5p mimic. NC- and miR-129-5p mimic-transduced ASCs were treated with TNF*α* plus IFN*γ* for 12 h, and cells were collected in TRIzol. mRNA levels were determined with quantitative RT-PCR. NC- and miR-129-5p inhibitor-transduced ASCs were treated with TNF*α* plus IFN*γ* for 60 min, and pStat1 expression was examined by western blot assay (b) and immunofluorescence staining (c). Scale bar: 200 *μ*m. NC- and miR-129-5p inhibitor-transduced ASCs were treated with TNF*α* plus IFN*γ* in the presence of fludarabine, mRNA levels were determined with quantitative RT-PCR (d), and iNOS protein levels were examined by western blot assay (e). ^∗^*p* < 0.05 and ^∗∗^*p* < 0.01.

To investigate how miR-129-5p may regulate the immunoregulatory function of ASCs, we transduced ASCs with lentiviral vectors containing miR-129-5p mimics or control lentivirus. GFP expression indicated high transduction efficiency (data not shown). As shown in Figure 6(a), when stimulated with TNF*α* and IFN*γ*, ASCs transduced with miR-129-5p mimics exhibited higher expression of TNF*α*, IFN*γ*, and IL-1*β* while exhibiting lower expression of iNOS. The Stat1 signaling pathway plays an important role in iNOS expression. Therefore, we next investigated the phosphorylation status of Stat1 in ASCs transduced with miR-129-5p inhibitor or control (NC). Western blot and immunofluorescence analyses demonstrated hyperphosphorylation of Stat1 (i.e., increases in pStat1) in ASCs transduced with miR-129-5p inhibitor (Figures 6(b) and 6(c)). When fludarabine, an inhibitor of Stat1 activation, was added, the expression of iNOS mRNA and protein in miR-129-5p inhibitor-transduced ASCs was significantly decreased (Figures 6(d) and 6(e)).

## 4. Discussion

ASC-based therapies for a variety of conditions ranging from tissue engineering to immunomodulatory applications have made significant advances in recent years [7–10]. A series of factors are known to be critical for ASC immunoregulation. The present study provides the first report on alterations of the immunomodulatory functions of ASCs by miR-129-5p. ASCs with miR-129-5p knockdown became more immunosuppressive, as shown by the reduced expression of proinflammatory factors and the increased expression of iNOS and NO production.

Like MSCs, the immunosuppressive function of ASCs is elicited by proinflammatory factors. In the present study, we show that miR-129-5p is induced by the inflammatory factors TNF*α*, IFN*γ*, and LPS in ASCs, similar to what has been observed for miR-155 and miR-150. Xu et al. reported that miR-155 was induced by inflammatory cytokines and inhibited the immunosuppressive capacity of MSCs by regulating the NF-*κ*B pathway thereby reducing iNOS expression and NO production [20]. We previously demonstrated that miR-150 overexpression in ASCs results in high NO production [19]. Here, our data revealed that miR-129-5p affected Stat1 activation and thus reduced iNOS expression and NO production in ASCs.

MSC-mediated immune regulation mainly occurs through paracrine effects by the production of soluble factors but can also occur through direct cell-cell interactions [28, 29]. Our previous studies revealed that SOCS1 [17] and the ubiquitin-modifying enzyme A20 [18] are also involved in the immunoregulatory functions of MSCs through the regulation of NO production and IL-10 secretion. In the present study, miR-129-5p knockdown in ASCs led to high iNOS expression and NO production with concurrent reduced inflammatory factor expression. The Stat1 signaling pathway plays an important role in iNOS expression [30]. ASCs with miR-129-5p knockdown that were treated with inflammatory factors exhibited hyperphosphorylation of Stat1 and thus high iNOS expression. The Stat1 signaling pathway also plays an important role in cytokine signaling and regulation of immune responses [31]. Activation of Stat1 was found to be related to high production of TNF*α* and IFN*γ* [32]. A balance of pStat1 and pStat3 has been documented in immunology [33]. Whether the reduced expression of TNF*α*, IFN*γ*, and IL-1*β* with Stat1 activation in ASCs with miR-129-5p knockdown is related to the balance of Stat3 activation requires further studies. When the Stat1 pathway was inhibited with fludarabine, the high expression of iNOS in miR-129-5p inhibitor-transduced ASCs was significantly decreased, which confirmed the involvement of Stat1 activation in ASCs with miR-129-5p knockdown.

## 5. Conclusions

The present study reveals that miR-129-5p regulates the immunosuppressive capacity of ASCs by targeting the Stat1 pathway and thus uncovers a previously undescribed role of miR-129-5p in regulating ASC immunomodulation. These novel insights into the mechanisms through which ASCs regulate immune responses may improve the clinical utility of these cells in immune-related diseases by targeting miR-129-5p, which could in turn help bring promising ASC therapies to patients in a safe and effective manner.

## Data Availability

Data related are available upon request.
